# Near‐infrared spectroscopy applications for high‐throughput phenotyping for cassava and yam: A review

**DOI:** 10.1111/ijfs.14773

**Published:** 2020-09-05

**Authors:** Emmanuel Oladeji Alamu, Ephraim Nuwamanya, Denis Cornet, Karima Meghar, Michael Adesokan, Thierry Tran, John Belalcazar, Lucienne Desfontaines, Fabrice Davrieux

**Affiliations:** ^1^ International Institute of Tropical Agriculture (IITA) Southern Africa Hub PO Box 310142 Chelstone, Lusaka Zambia; ^2^ International Institute of Tropical Agriculture (IITA) PMB 5320, Oyo Road Ibadan Oyo State Nigeria; ^3^ National Crops Resources Research Institute NaCRRI P.O Box 7084 Kampala Uganda; ^4^ CIRAD UMR AGAP Montpellier F‐34398 France; ^5^ Univ. Montpellier CIRAD INRA Montpellier SupAgro France; ^6^ UMR Qualisud University of Montpellier CIRAD Montpellier SupAgro University of Avignon University of La Réunion 73 rue JF Breton Montpellier 34398 France; ^7^ The Alliance of Bioversity International and the International Center for Tropical Agriculture (CIAT) CGIAR Research Program on Roots Tubers and Bananas (RTB) Apartado Aéreo 6713 Cali Colombia; ^8^ Centre de recherche Antilles‐Guyane INRAe UR 1321 ASTRO Agrosystèmes tropicaux Petit‐Bourg France

**Keywords:** Cassava, yam, quality traits, NIRS, high throughput, hyperspectral imaging

## Abstract

The review aimed to identify the different high‐throughput phenotyping (HTP) techniques that used for quality evaluation in cassava and yam breeding programmes, and this has provided insights towards the development of metrics and their application in cassava and yam improvements. A systematic review of the published research articles involved the use of NIRS in analysing the quality traits of cassava and yam was carried out, and Scopus, Science Direct, Web of Sciences and Google Scholar were searched. The results of the review established that NIRS could be used in understanding the chemical constituents (carbohydrate, protein, vitamins, minerals, carotenoids, moisture, starch, etc.) for high‐throughput phenotyping. This study provides preliminary evidence of the application of NIRS as an efficient and affordable procedure for HTP. However, the feasibility of using mid‐infrared spectroscopy (MIRS) and hyperspectral imaging (HSI) in combination with the NIRS could be further studied for quality traits phenotyping.

## Graphical abstract

NIRS technique for high throughput phenotyping for cassava and yam.

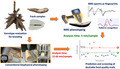

## Introduction

Breeding programmes need to screen large numbers of genotypes for agronomic, nutritional quality and end‐product quality traits to select the best ones for the next breeding and selection cycles. However, to consistently assess end‐product quality, it is crucial to increase the understanding of crop reactions to different environments and management practices and genotype by environment by management interactions. High‐throughput phenotyping (HTP) methods that efficiently predict end‐product quality traits would facilitate economic and timely inclusion of end‐user traits in the selection process of large breeding populations. Cassava and yam are essential roots and tuber crops that are commonly grown in the tropic and subtropical parts of the world. Roots and tuber crops serve as a significant source of dietary carbohydrates. For instance, cassava plays a vital role as stable for over 500 million world population due to its high carbohydrate content (Blagbrough *et al*., 2010). Yam, on the other hand, is also a starch‐rich tuber crop which plays pivotal nutritional and cultural roles in the West African region (Ferede *et al*., 2010; Alamu *et al*., 2014 ). Yam tubers have specific bioactive components such as polyphenols, diosgenin, vitamins,carotenoids and tocopherols (Bhandari *et al*., 2003; Ferede *et al*., 2010; Alamu *et al*., 2016).

These HTP procedures can be merged with evaluation methods already in use for value chain traits such as yield, pest resistance, processing ability and market acceptability. Among the HTP procedures available for cassava and yam improvement breeding programmes, near infrared spectroscopy (NIRS) has excellent potential for simultaneous prediction of various quality traits (Lebot & Malapa, 2013; Sánchez *et al*., 2014; Belalcazar *et al*., 2016; Ikeogu *et al*., 2017; Abincha *et al*., 2020). It has proven effective for the prediction of dry matter, carotenoids and cyanogenic potential in raw cassava roots (Belalcazar *et al*., 2016), and dry matter and carotenoids in fresh sweet potato. For other traits, such as individual sugars, NIRS had better accuracy in dried and milled potato and sweet potato samples compared to raw materials. NIRS application is associated with little or no sample preparation, cost‐effectiveness and time‐effectiveness and high sample throughput in comparison with conventional methods. Also, its correlation to high end and highly reproducible procedures such as chromatographic techniques has been established, making it an easy tool to apply in settings where alternative sample handling options are time‐consuming and expensive (Jaramillo *et al*., 2018; Abincha *et al*., 2020).

In addition to the use of NIRS, spectral imaging was suggested as a tool for HTP for cassava and yam. This type of imaging uses multiple bands across the electromagnetic spectrum. The spectral imaging technique is a combination of NIRS and imaging where spectral information is collected on an array of pixels in a sample, resulting in the simultaneous acquisition of spatial images and spectral information (Sun, 2009). Spectral imaging can be divided into two main subcategories: multispectral imaging (MSI) which captures a small number of spectral bands, typically three to fifteen, using different filters and illuminations and hyperspectral imaging (HSI) which combines spectroscopy and digital photography. A hyperspectral camera captures hundreds of wavelength bands which can be interpreted as a complete spectrum, for each pixel. This nearly continuous spectrum, made of narrow and contiguous spectral bands, is vital to optimising the use of the information, making possible the simultaneous quantitative prediction of chemical and physical properties and their spatial distribution (Sun, 2009).

In this review, we identified the different high‐throughput procedures that have been used in the characterisation of cassava and yam germplasms. Such knowledge is critical to provide insights towards the development of metrics and their application in cassava and yam improvement. Importantly, we wanted to verify the application of these procedures in breeding programmes, forming a basis for their wide adoption.

## NIRS and HSI techniques for high‐throughput phenotyping for cassava

### Spectroscopic techniques

One of the most common spectroscopic techniques used in cassava‐based studies was NIRS. The technique measures the interactions between electromagnetic radiation and vibrational properties of chemical bonds (Cozzolino, 2015). The visible (VIS) spectra cover the range from 380 nm to 780 nm, which returns mainly information on colour due to pigments present in the sample. NIR refers to the 780–2500 nm part of the electromagnetic spectrum and is more useful for quantitative analysis of complex mixtures (Sun, 2009). The mid‐infrared (MIR), approximately 2500 nm–25 000 nm (4000–400 cm^−1^), was also suggested in some studies as relevant in the understanding of the fundamental vibrations and associated rotational–vibrational structure of food‐based products. MIRS is thus explicitly used to determine the chemical functional groups of a sample in both qualitative and quantitative ways (Sun, 2009).

The utilisation of NIRS in cassava breeding is advancing the research, farming and industrial agendas of cassava improvement and can be harnessed in a significant range of applications. These include breeding selection for high‐quality cassava products, but also improving the safety of cassava products, detection of adulterants, detection of altered metabolism in cassava plant or tissue and detection of changes in already developed products (Table 1).

**Table 1:** Summary of HTP techniques used for quality traits evaluation in roots and tubers

Technique	Quality trait	Process and product	Chemometrics and software	Instrument	References
Raman spectroscopy and Surface Enhanced Raman Spectroscopy	Cyanide Potential	Leaves extracts (cassava)	Based on CN bond Peak area measurement with standard	Dual‐laser LabRaman Infinity‐Jobin ‐Yvon	Thygesen *et al*. (2004)
NIRS	Starch (*R* ^2^ = 0.90, SECV = 0.32) Proteins (*R* ^2^ = 0.62, SECV = 0.20)	Raw ground (potato)	MPLS Winisi‐FOSS	6500‐FOSS	Haase (2006)
NIRS	Physiochemical quality	Starch (sweetpotato)	MPLS WinIsis‐FOSS	NIRSystem 5000‐FOSS	Lu *et al*. (2006)
SEM, X‐ray diffraction and Fluorescence, UV‐Vis, IR spectroscopy	Residual Cyanide	Different processing modes (cassava)	Spectra and Image processing sofware ‐Not specified‐	S‐4700‐ Hitachi, D5005‐Siemens, Cary bio300‐Varian, Eclipse‐Varian Spectrum One‐Perkin‐Elmer,	Phambu *et al*. (2007)
NIRS	Pasting properties, apparent amylose and gel texture	Whole grain and Flour (rice)	MPLS Winisi‐FOSS	NIRSystem 5000‐FOSS	Bao *et al*. (2007)
FT‐NIRS	Rheological and Chemical properties	Starch (potato and maize)	PLS and PCA Uncrambler‐Camo	Antaris I‐Thermo Fisher Scientific	Schrampf & Leitner (2010)
NIRS	Processing quality	Raw ground (potato)	PLS Winisi‐FOSS	6500‐FOSS	Haase (2011)
NIRS	Total N and minerals, plus starch, sugars and cellulose	Oven dried Chips (cassava and others)	PLS XLSTAT‐Addinsoft, GRAMS‐Thermo Fisher Scientific	LabSpecPro, ASD	Lebot *et al*. (2013)
NIRS	Review	Raw (potato)	Review	Review	Lopez *et al*. (2013)
FT‐NIRS	Adulteration	Root powder (lotus)	SIMCA and PLSCM Matlab‐Mathworks	Tensor37 FTI‐Bruker	Xu *et al*. (2013)
NIRS	Quality parameters	Whole Grain and flour (rice)	PLS and PCA Uncrambler‐Camo	DA7200 diode array‐Perten Instrument	Sun *et al*. (2014)
NIRS	TCC (*R* ^2^ = 0.86, SECV = 284) HCN (*R* ^2^ = 0.89, SECV = 1.34)	Raw, Ground (cassava)	MPLS, Winisi‐FOSS	6500‐FOSS	Sánchez *et al*. (2014)
FTIR microspectroscopy	Treatment of plant tissue with stress factor	Leaves tissue (Cassava)	Identification of Spectral and biochemical changes‐Cytospec 1.3.4‐Cytospec Inc.	FTRIR‐Vertex70 and IR microscope ‐Bruker	Thumanu *et al*. (2015)
FT‐NIRS	Adulteration	Starch (Kudzu)	PLSCM Matlab‐Mathworks	Tensor37 FTI‐Bruker	Xu *et al*. (2015)
HPLC with UV‐VIS Spectroscopy	Genotypes classification	Raw (Cassava)	PCA, ANOVA, AHC R‐Core Team Chemospec,HyperSpec and ggplot2	Gold Spectrum lab53, Bel photonics and LC‐10A, Shimadzu	Moresco *et al*. (2015)
NIRS	TBC *R* ^2^ = 0.48 DM *R* ^2^ = 0.09	Raw, Ground (Cassava)	MPLS Winisi‐FOSS	6500‐FOSS	Belalcazar *et al*. (2016)
NIRS	TCC (*R* ^2^ = 0.94, SECV = 2.63) DM *R* ^2^ = 0.96, SECV = 1.20	Raw, Ground (Cassava)	Local regression Winisi‐FOSS	6500‐FOSS	Davrieux *et al*. (2016)
NIRS	TCC (*R* ^2^ = 0.90, SECV = 0.63) TBC, DM (*R* ^2^ = 0.91, SECV = 0.90)	Raw, Intact and Mashed (Cassava)	MPLS Winisi‐FOSS	QualitySpec Trek‐ASD	Ikeogu *et al*. (2017)
NIRS	Maleic acid (RMSEP = 0.19)	Starch (cassava)	OCPLS, LS‐SVM Matlab‐Mathworks	Nicolet 6770 FTIR ‐Thermo Fisher Scientific Inc	Fu *et al*. (2017)
HSI‐NIRS	Adulterations (*R* ^2^ = 0.98, SECV = 0.026)	Flour (cassava)	FMCIA‐PLS Matlab‐Mathworks	CCD camera Xeva 992‐Xenics Infrared Solutions	Su & Sun (2017)
iCheck Carotene, and UV‐VIS, NIR spectroscopy	TCC (*R* ^2^ = 0.99) trans‐BC (*R* ^2^ = 0.98)	Raw, Ground (Cassava)	MPLS Winisi‐FOSS	μQuant‐BioTek Instruments, iCheck Carotene‐BioAnalyt GmbH, 6500‐FOSS, LabSpec4‐ASD	Jaramillo *et al*. (2018)
NIRS	TBC (*R* ^2^ = 0.86, SECV = 0.77) TCC (*R* ^2^ = 0.81, SECV = 1.25)	Raw, sliced (Cassava)	MPLS Winisi‐FOSS	XDS‐FOSS	Alamu *et al*. (2019a)

AHC, ascending hierarchical classification; ANOVA, analysis of variance; FMCIA, first‐derivative and mean centring iteration algorithm; FT‐NIRS, Fourier transform near‐infrared spectroscopy; LS‐SVM, least squares support vector machine; MPLS, modified partial least squares regression; OCPLS, one‐class partial least squares; PCA, principal component analysis; PLS, partial least squares; PLSCM, partial least squares class model; *R* ^2^_,_ correlation coefficient in cross validation; RMSEP, root‐mean‐square error of prediction; SECV, standard error of cross validation; SEM, scanning electron microscopy; SIMCA, soft independent modelling of class analogy.

Raw, ground or mashed = blended fresh root; raw intact = unbroken fresh root; raw sliced = sliced fresh root; flour = dried and milled with grinder; starch = extracted starch from the fresh root.

Furthermore, in a bid to support the genetic improvement of crops such as cassava, large databases of biophysical and NIRS data are developed, to establish robust calibrations based on diverse sample sets (Lebot *et al*., 2013). Specific references such as Davrieux *et al*. (2016) and Shen *et al*. (2019) provide procedures on how large databases can be handled using several methods for the development of accurate predictions for various traits.

### Target constituents and physical properties in cassava

The reviewed studies on cassava were summarised according to the analytical techniques used, the sample preparation procedure and the chemometric methods applied (Table 1). Regarding fresh or processed cassava, most of the NIRS investigations reported quantification of chemicals constituents, including total carotenoids content (TCC), total beta‐carotene (TBC), dry matter (DM), hydrogen cyanide (HCN), starch and sugars. Some of the papers reported safety and adulteration concerns observed or expected in the cassava value chain such as adulteration of cassava flour. The primary scanning mode is diffuse reflectance and the principal chemometric methods selected and applied are partial least square (PLS) and principal component analysis (PCA). The technique is used mainly in understanding the chemical constituents (carbohydrate, protein, vitamins, minerals, carotenoids, moisture, starch and fat) for quality control or HTP. Also, physical attributes such as specific gravity, skin colour and texture and quality aspects related to the processing of the tubers have been determined using NIRS. The universal application of this tool and its ability to provide relevant information that defines different product profiles explains much about its attractiveness.

### NIRS studies for high‐throughput phenotyping for cassava root quality

NIRS is mainly used in understanding changes in nutritional parameters of cassava roots. Indeed, several breeding programmes and several crops are already applying NIRS in the breeding of nutritious varieties (Lebot & Malapa, 2013; Lebot *et al*., 2013; Alamu *et al*., 2019). In most cases, NIRS is being used to evaluate a range of traits in a breeding population with comparisons among these traits supporting the selection of superior varieties (Lebot *et al*., 2009; Tumwegamire *et al*., 2011; Lebot *et al*., 2013). Besides, the use of NIRS in the evaluation of location‐based differences arising in populations concerning traits such as dry matter, starch, sucrose, β‐carotene content and minerals has been demonstrated in sweet potato (Tumwegamire *et al*., 2011) and is highly relevant in cassava too. With such a broad range of applications, NIRS for phenotyping is also finding new applications in the assessment of genetic correlations, such as genome‐wide association studies (GWAS) and genomic predictions (Ikeogu *et al*., 2019).

Phambu *et al*. (2007) investigated various techniques (fluorescence, infrared spectroscopy, scanning electron microscopy, UV‐visible spectroscopy and X‐ray diffraction) to monitor the effect of post‐harvest processing on the residual cyanogens in cassava roots. Two types of infrared methods were tested, using a Spectrum One spectrometer (Perkin‐Elmer): (i) For transmission spectra, samples were prepared as KBr discs with 10% w/w of each sample; (ii) for ATR‐IR spectra, an ATR DuraVision accessory was used to record the spectra of pure samples, without any preparation. The authors demonstrated that soaking and sun‐drying reduced cyanogens while boiling did not significantly affect the chemical composition of cassava. The infrared technique (4000–6000 cm^‐1^) was able to detect residual cyanide at relatively low concentrations. Freitas *et al*. (2020) applied NIRS techniques to monitor the effects of frogskin disease in cassava. They demonstrated early detection of the disease, with the advantages of more accurate detection and lower cost compared to conventional methods. In such cases, NIRS techniques are based on understanding the changes in biochemical parameters in the affected parts of the cassava plant or the response of the plant to an environmental factor (Thumanu *et al*., 2015).

Other applications of NIRS on cassava were on the biochemical components' quantification (Total carotenoids – TCC, Total beta‐carotene – TBC and Drymatter – DM). Ikeogu *et al*. (2017) developed models for DM, TBC, TCC quantification, with an accuracy expressed as standard error cross validation (SECV) = 0.9%, 1.6% and 2.1%, respectively. These models based on PLS (partial least square) regression were developed on crushed fresh cassava by using a portable visible–near‐infrared (VIS‐NIR) spectrometer (QualitySpec Trek: S‐10016, ASD; Longmont, USA) and were more accurate than those based on spectra of intact fresh cassava samples. This study was complementary to studies related to the quantification of TCC and TBC in fresh cassava samples by Sánchez *et al*. (2014); Belalcazar *et al*. (2016); Davrieux *et al*. (2016) and Moresco *et al*. (2015). The accuracy of the models developed by Davrieux *et al*. (2016) enables the use of NIRS as HTP of cassava genotypes for high TBC and TCC contents. The models are based on an extensive database (6026 samples) together with a local PLS regression approach. Notably, the standard error of prediction (SEP) values were 1.38% and 1.03%, respectively, for TBC and TCC, suggesting a wide range of applications in different populations. For the first time, Fu *et al*. (2017) quantified maleic acid content (RMSEP (root‐mean‐squared error of prediction) = 0.026%) to detect adulteration in fraud labelling of cassava starch by manufacturers or traders. The study, therefore, showed the application of NIRS in product quality assessments. Such rapid assessments are critical in the marketing and use of cassava for various purposes.

Only one article was published on HSI applied to cassava. Su & Sun (2017) aimed to detect cassava flour adulterants in Irish organic wheat flour (OWF). Hyperspectral images (900–1700 nm) of OWF samples with a series of adulteration percentages were collected. Partial least squares regression (PLSR) and principal component regression (PCR) were employed for quantitative analysis. The best prediction model of adulteration was developed using a first‐derivative and mean centring iteration algorithm (FMCIA).

Table 1 provided the information on the NIRS applications, the sample presentation, the chemometric methods and software used, and the type or brand of the instrument used. Different NIRS instruments are available in the market and are used for different applications and traits. The most commonly used instrument, across traits and cassava improvement programmes, was FOSS instruments, followed by ASD quality Spec and ASD LabSpec Pro. The types of sample and the place of analysis (e.g. laboratory or field) also need to be considered: A benchtop instrument offers better performance when the laboratory is located near the experimental fields. On the other hand, portable NIRS instruments offer flexibility if phenotyping must be conducted away from the laboratory, or at multiple locations within a country or region. Among all the reviewed studies, these considerations, the ease of use of the instrument and operational costs were not discussed. However, compatibility challenges remain in the establishment of the crosstalk between different instruments. It is an issue mainly when assessments between different laboratories or breeding programmes must be performed.

Lebot *et al*. (2013) suggested the possibility of using NIRS across crops and different traits as a cost‐cutting measure for field evaluation of genotypes. Such improvement in the efficiencies is essential, especially in African breeding programmes. However, this should be backed up by data management options that allow each improvement programme to utilise their data in the best way possible.

Most of the studies reported two main traits (DM and TBC), but the assessment of other traits needs to be considered to utilise the potential of NIRS technologies fully. Some of these traits include those that affect the cooking properties of the cassava root, industrial‐based traits that allow for easy processability and how cassava‐based products interact with other materials especially in areas where cassava is not consumed alone. The changes in traits after processing could also be considered (Table 2).

**Table 2:** Proposals for future high‐throughput phenotyping of cassava

Trait	Current methods	Recommended HTP	References
Determination of Amylose, Amylopectin, Starch and Granule Structure	Iodine – colorimetric method Enzymatic and acid‐based hydrolyses Polarised light microscopy, X‐ray diffraction (XRD) and FTMIR spectroscopy	NIRS (FOSS or Brucker)	Hong *et al*. (1996), Katayama *et al*. (1996) and Campbell *et al*. (1999)
Gelatinisation, Pasting Properties and Retrogradation of Starch	RVA pasting profile DSC	FT‐NIR‐spectrophotometer (Thermo Fisher Scientific Inc) NIR Spectroscopy (FOSS or Brucker)	Lu *et al*. (2006), Zardetto and Rosa (2006), Lu and Sheng (1990)
Monitoring of Starch Gelatinisation and Processing	Process analytical technologies (PAT), scanning electron microscope (SEM), FTIR, XRD and DSC methods	NIR spectroscopy (FOSS or Brucker), FT‐Raman spectroscopy (Dual‐laser LabRaman Infinity‐Jobin ‐Yvon)	Cogdill *et al*. (2004), Jorgensen *et al*. (2004), Robert *et al*. (1999)
Fresh or Boiled cassava surface properties	No methods stated	Light back‐scattering imaging (Hyper spectral imaging‐HIS; Xenics Infrared Solutions)	Adebayo *et al*. (2016)

The use of NIRS in predicting root and tuber product quality in cassava and yams is a feasible option that requires the development of relevant procedures that apply to quality control parameters demanded. Lebot et al. (2009), provided an extensive overview of the use of NIRS in the prediction of quality control parameters in a range of root and tuber crops. The main quality control parameters considered included starch, total sugars, cellulose, total nitrogen and ash (total minerals) contents. Predictive accuracy ranging for starch, sugar and nitrogen ranged from 86% to 93% much as the prediction for cellulose was not possible. From this study, it was observed that NIRS as a low‐cost technique could be adapted to quality control schemes for screening many samples with high accuracy. Relatively similar studies have been undertaken for taro (Lebot *et al*., 2011a, 2011b), potatoes (Huang *et al*., 2018), sweet potato (Lu *et al*., 2006) and even for root and tuber crop‐based flour blended with cereal flour (Huang *et al*., 2018) or consumptive products such as bread (Wang *et al*., 2019). Su & Sun (2017) explored Spectral imaging for quantitative detection of Irish organic wheat flour adulterated with cassava flour and cornflour. Furthermore, they reported that spectral imaging integrated with multivariate analysis has the potential to authenticate the admixtures in specific wheat flour in the range of 3–75% (w/w).

## NIRS studies for high‐throughput phenotyping for yam tuber quality

Few studies have used NIRS to characterise yam (*Dioscorea* spp.) tubers (Table 3). Overall, despite the large number of species investigated, only a few accessions were scanned (i.e. 320 *D. alata,* 223 *D. opposite,* 182 *D. zingiberensis,* 153 *D. rotundata,* 39 *D. dumetorum,* 24 *D. bulbifera,* 15 *D. cayenensis,* 14 *D. esculenta,* 9 *D. transversa,* 7 *D. nummularia,* 6 *D. prahensilis* and 3 *D. mangenotiana*). Increasing the number of scanned accessions should improve future model performances to predict the chemical composition and quality traits (Table 4), both in terms of accuracy and robustness. However, to tackle more accessions, sample preparation has to be simplified and sped up. So far, all the studies but Kwon *et al*. (2015) recorded NIRS spectra on the dried product (flour), which is tedious and time‐consuming due to the steps of drying and milling the tubers. Kwon *et al*. (2015) worked on freeze‐dried samples, which is also time‐consuming.

**Table 3:** Summary of near‐infrared spectroscopic studies for the quality evaluation of yams (*Dioscorea* spp.)

Study	Product	Model	Software used	*N_c_*	*N_v_*	Instrument	Range	Sampling interval
Alamu *et al*. (2019b)	Flour	MPLS	ISI scan software	126	37	FOSS (XDS)	400–2500 nm	0.5 nm
Lebot *et al*. (2009); Lebot *et al*. (2013); Lebot & Malapa (2013)	Flour	PLS	GRAMS/AI & PLSPlus/IQ	210	50	ASD (LabSpecPro)	350–2500 nm	2 nm
Bai *et al*. (2013)	Powder	PLS	TQ software	77	17	Nicolet (6700 FTIR)	12 000–4000 cm^−1^	8 cm^−1^
Xie *et al*. (2013)	Powder	PLS	not specified	71	17	Nicolet (6700 FTIR)	12 000–4000 cm^−1^	8 cm^−1^
Zhuang *et al*. (2015)	Powder	PLS & SVM	not specified	73	35	Hitachi (U–4100)	12 500–4000 cm^−1^	10 cm^−1^
Kwon *et al*. (2015)	Freeze‐dried	PLS	OPUS program & R	122	61	Bruker (not specified)	4000–400 cm^−1^	4 cm^−1^

Nc, number of samples used for the calibration step. Nv, number of samples used for the validation step.

**Table 4:** Targeted tuber composition and prediction accuracy in yam near‐infrared spectrometry studies

Analyte	Study	Unit	Mean	Min	Max	*N*	SD	*R* ^2^	RPD
Moisture	Alamu *et al*. (2019b)	%	6.94	3.79	9.75	163	1.67	0.80	2.24
Total sugar	Zhuang *et al*. (2015)	%	75.9	74.0	78.7	108	0.79	0.99	4.17
Starch	Alamu *et al*. (2019b)	%	50.7	25.1	66.1	163	10.32	0.42	1.29
	Lebot & Malapa (2013)	%	77.6	58.8	90.4	260	5.11	0.84	2.86
Polysaccharide	Zhuang *et al*. (2015)	%	23.2	15.2	35	108	1.20	0.96	2.74
Soluble sugar	Alamu *et al*. (2019b)	%	5.0	2.1	9.0	163	1.15	0.31	2.76
	Lebot & Malapa (2013)	%	2.4	0.1	18.3	260	3.16	0.86	4.05
Amylose	Alamu *et al*. (2019)	%	32.5	18.8	44.4	163	4.82	0.27	1.14
	Lebot & Malapa (2013)	%	54.5	37.3	68.9	125	5.19	0.18	1.40
Cellulose	Lebot & Malapa (2013)	%	4.53	0.1	11.98	260	2.45	0.10	1.94
Crude fibre	Alamu *et al*. (2019b)	%	2.16	1.22	3.52	163	0.44	0.68	0.62
Protein/nitrogen	Alamu *et al*. (2019b)	%	6.8	3.3	9.7	163	1.57	0.69	1.87
	Lebot & Malapa (2013)	%	9.9	4.4	21.0	260	2.64	0.88	3.64
Fat	Alamu *et al*. (2019b)	%	0.31	0.05	1.73	163	0.19	0.14	0.70
Minerals/ash	Alamu *et al*. (2019b)	%	3.58	1.96	6.26	163	0.74	0.68	3.47
	Lebot & Malapa (2013)	%	4.63	1.58	8.14	260	1.16	0.22	1.69
Phytate	Alamu *et al*. (2019b)	mg 100 g^−1^	1.17	0.33	2.44	163	0.42	0.29	1.15
Tanin	Alamu *et al*. (2019b)	mg 100 g^−1^	2.04	0.05	9.91	163	1.93	0.50	1.58
Dioscin	Xie *et al*. (2013)	%	0.23	0.09	0.40	88	n.p.	0.92	n.p.
	Kwon *et al*. (2015)	ppm	0.16	0.00	1.10	183	0.29	0.72	n.p.
Diosgenin	Bai *et al*. (2013)	%	1.36	0.62	2.56	94	0.36	0.96	2.84
Flavonoid	Zhuang *et al*. (2015)	%	0.21	0.13	0.28	108	0.011	0.97	2.79

Mean, measured average value. Min, analyte minimum measured value. Max, maximum measured value. N, number of observations. SD, standard deviation of reference values. *R* ^2^, coefficient of determination of the regression between predicted and measured values during the validation step. RPD, the ratio of performance to deviation. (n.p.: not provided).

In contrast, working on fresh, intact tubers, in the laboratory or straight in the field with a portable spectrometer, has the potential to speed up spectra acquisition, hence taking full advantage of NIRS as an HTP tool for rapid screening and selection in breeding programmes. Working on fresh tubers also avoids chemical or structural modifications linked to drying or milling. Of course, special attention must be paid to sampling in order to consider the heterogeneity of the product.

Although most of the reviewed studies worked with the same product (i.e. yam flour), each of them used a different spectrometer, with variable spectral range and sampling interval. Given the scarcity of available studies, the impossibility of transferring a predictive model from one spectrometer to another emphasises the need to develop spectrum normalisation and interoperability. Moreover, most studies resort to partial least square regression and commercial software to carry out the multivariate analysis. Only Kwon *et al*. (2015) tested support vector machine regression using open‐source R statistical software. Because relationships between spectral values and the analyte may not be linear, the investigation in non‐linear techniques, such as deep learning algorithms, may improve the performance of the prediction significantly. Many open‐source programming languages (e.g. Python, R, octave) offer powerful libraries (e.g. Keras, caret) and platform (e.g. TensorFlow) dedicated to regression tools and deep learning algorithms.

Table 4 summarises the analytes targeted by NIRS studies on yam tuber quality: carbohydrates (i.e. total sugar, polysaccharide, starch, amylose, soluble sugar and cellulose), protein, fat, minerals, crude fibre and secondary compounds (i.e. tannin, flavonoid and phytate). Except for amylose, the values of the studied constituents cover the range well and are consistent with the reference values in the literature (Polycarp *et al*., 2012). Amylose content found in 2013 by Lebot & Malapa (37.3% to 68.9%) are far over the reference values (24.3% to 38.1%) and should be taken with caution. Published studies offer accurate prediction (*R* ^2^ > 0.8) for moisture, total sugar, starch, polysaccharides, soluble sugar, protein, total nitrogen, dioscin, diosgenin and flavonoids. On the other hand, the prediction of amylose, cellulose, crude fibre, tannin, fat, ash and phytate contents proved less reliable. As already stated, alternatives to PLS may allow improving performances thanks to their ability to manage nonlinearity and to identify spatial relationships between spectrum features (peak patterns) and wavelengths (e.g. Convolutional Neural Network, Bidirectional Long Short‐Term Memory Neural Network). However, some traits may not be effectively predicted with NIRS. Near‐infrared Fourier transform and Raman spectroscopy (NIR FT‐Raman) allow studying the molecular structure. Liao *et al*. (2004) used NIR FT‐Raman to analyse the compositional and conformational properties of yam (*D. alata* and *D. japonica*) proteins. Mid‐infrared spectroscopy (MIRS) can also gather information on the molecular content of products. Zhuang *et al*. (2015) demonstrated that MIRS provides a better prediction of flavonoid and polysaccharide content, while NIRS offers a better prediction for total sugar content.

Computer vision systems (CVS) are used to improve the characterisation of the colour of fruit and vegetables (Mendoza *et al*., 2004). Because the colour of yam tubers is heterogeneous in time (oxidation) and space (radial and longitudinal gradient), the use of image analysis may improve actual qualitative (visual observation) or quantitative (colourimeter and chromameter) practices, by offering the ability to assess the variability of colour and oxidation and to quantify colour precisely. Moreover, this technique has the potential to bring more information (e.g. tuber shape and skin thickness) linked to useful quality traits (e.g. peeling yield, easiness of harvest). Some preliminary studies are ongoing within the framework of the RTBfoods project (https://www.cirad.fr/en/news/all‐news‐items/press‐releases/2018/rtbfoods).

The texture of cooked root and tuber crops is recognised as a primary determinant of consumer acceptability of new varieties (Goddard *et al*., 2015). However, all reviewed yam studies focused on the quantification of biochemical constituents. Only a few included texture and functional traits such as friability and mouldability (i.e. the capacity to form a dough while pounded). These traits are nevertheless essential for evaluating the phenotype suitability for specific product applications (e.g. pounded or boiled yam), calling for more studies on the subject.

## Limitations of NIRS in phenotyping cassava‐ and yam‐based quality traits

Despite the usefulness of NIRS as a phenotyping tool, there are several limitations related to its reproducibility and the availability of data analysis software compatible with all data manipulation requirements. Successful utilisation of NIRS relies heavily on the development of standard data analysis procedures and statistical software which may be lacking or unaffordable in some breeding programmes. Open‐source programming languages offer the possibility to develop customised analyses packages, including management of outliers, pretreatments of data and calibration steps altogether (Belagiannis *et al*., ; Cui & Fearn, 2018). While the optimum combination of these three steps may depend on the analyte or quality trait under study, the procedure to identify it may be standardised. Usual methods (e.g. multi‐scatter correction, local PLS) may be combined with recent deep learning techniques through model ensembling. The development of such a customised package is still ongoing. Meanwhile, many tools are already available to manage NIRS data workflow (e.g. ChemFlow, TensorFlow).

At the scale of a multi‐partner network focused on applying NIRS to under‐researched crops such as roots and tubers, the ability to share data and calibration models is crucial. This question requires mastering the production of data (sample preparation and spectra acquisition), the sharing of data (common ontology and database management) and the transferability of prediction models (standardisation of spectra between spectrometers). Under the RTBfoods project, Standard Operating Protocols (SOP) ensure the standardisation of sample preparation and data production. Once generated, data and their associated metadata are shared using standard ontology terms (e.g. https://www.cropontology.org/ontology/CO_343/Yam) and database management tools such as Yam Base (https://yambase.org/) and Cassava Base (https://www.cassavabase.org/). Managing interoperability is still an open question. However, recent studies offer promising results based on deep learning algorithms (Chatzidakis & Botton, 2019).

Before embarking on the use of NIRS for quality traits phenotyping, a breeding programme needs to be sure that adequate resources will be available to effectively exploit the technology (Li *et al*., 2014). Another limitation in applying NIRs and other spectroscopic techniques for screening applications is managing the large volume of data that can be generated from spectral signature (Li *et al*., 2014). HTP techniques such as multispectral or hyperspectral imaging cameras are relatively expensive, hence limiting their adoption (Li *et al*., 2014). Nevertheless, they are essential if breeding programmes are to answer to the expectations of consumers and other stakeholders in the cassava market chain. HTP also requires reporting not only large data sets, but also the associated meta‐information concerning experimental protocols, data management system and integration with modelling (Fiorani, & Schurr, 2013).

## Conclusions

The review confirmed that NIRS could rapidly predict moisture, total sugar, starch, polysaccharides, soluble sugar, protein, total nitrogen, dioscin, diosgenin and flavonoids. Moreover, total nitrogen, starch and sugar concentrations could be predicted with a single calibration applied to five different root crop species (cassava, yam, sweet potatoes, taro and cocoyam) and across a wide range of varieties. On the other hand, the prediction of amylose, cellulose, crude fibre, tannin, fat, ash and phytate contents proved to be less reliable. NIRS complements, and in some cases can replace, complex laboratory procedures for quality evaluation, generally with the advantages of minimal sample preparation and rapid analysis. The publications reviewed underlined the potential of NIRS for high‐throughput screening and quality control of cassava and yam genotypes and samples. However, robust models based on large data sets are still needed to precisely predict quality attributes for new samples, especially for breeding purpose. The data sets should be obtained from different locations, growing conditions and post‐harvest conditions in order to cover the variability of the trait to be quantified/characterised.

Spectra imaging devices such as multispectral spectra (MSI) and hyperspectral imaging (HIS) are emerging HTP devices which combine spatial imaging and spectral information. Their advantage is to provide a simultaneously quantitative prediction of chemical and physical properties and information on the spatial distribution of these traits across the roots cross sections. Computer vision systems (CVS) could also be used as HTP method to improve the characterisation of colour for yam tubers and cassava roots. CVS can measure the variability of colour and oxidation across a sample, and quantify colour precisely, and provide information such as tuber shape and skin thickness, linked to useful quality traits (e.g. peeling yield, ease of harvest).

## Future Challenge

However, despite some successful applications, specific challenges remain related to reproducibility. Affordability of the instruments and availability of suitable data analysis software is also of concern. These challenges call for concerted efforts by breeding programmes to increase adoption of HTP techniques for quality traits evaluation. Manufacturers and software developers of high‐throughput devices should provide extensive support on the purchase and during operations. RTB research programmes then need to translate the quality traits of interest, as identified by the user and consumer preferences studies, into measurable variables or indirectly correlated variables, and finally to develop a strategy for calibration with HTP techniques. This strategy includes the choice of non‐destructive HTP technique, the sampling method, sample preparation and presentation, measurement protocol and the choice of chemometrics methods.

For quality traits that cannot be assessed by NIRS prediction, a medium‐throughput product profiling (MTPP) approach can be developed, for example, amylose content classification, rapid texture evaluation, etc. First, NIRS can be used to pre‐screen genotypes into different classes of the trait to evaluate (instead of quantitative evaluation). Then, MTPP is applied only on the genotypes belonging to the most promising class.

Most HTP methods published so far on cassava and yam phenotyping analysed biochemical composition. Future studies may focus on also predicting quality traits of end‐products, that are relevant for varietal adoption by end‐users and consumers. This is potentially more complex since the quality of end‐products derives not only from the initial composition of the roots but also from the chemical reactions during processing, the latter of which NIRS and other HTP techniques may not be able to capture. Several studies in the framework of the RTBfoods project (https://www.cirad.fr/en/news/all‐news‐items/press‐releases/2018/rtbfoods) are ongoing towards this end. If successful, these approaches would markedly increase the usefulness and field of application of HTP techniques in RTB breeding programmes. Initial results indicate that textural softness and water absorption during boiling of cassava (Tran *et al*., 2021) can be predicted from the NIRS spectra of the fresh roots, from which cooking time can also be estimated. In a separate study, mid‐infrared spectroscopy (MIRS) of cell wall extracts enabled classifying cassava genotypes into two groups according to their cooking time: short (<30 min) or long (>30 min), with prediction accuracy of 80.3% and 69.6%, respectively. This link between cooking time and cell walls suggest that cell wall components, possibly pectins (Eggleston & Asiedu, 1994), play an important role in determining the texture of cassava products.

## Author contribution

**Emmanuel Oladeji Alamu:** Conceptualization (equal); Writing‐original draft (lead); Writing‐review & editing (lead). **Ephraim Nuwamanya:** Conceptualization (supporting); Writing‐original draft (lead); Writing‐review & editing (supporting). **Denis Cornet:** Conceptualization (supporting); Writing‐original draft (supporting); Writing‐review & editing (equal). **Karima Meghar:** Writing‐original draft (supporting); Writing‐review & editing (supporting). **Michael Adesokan:** Conceptualization (supporting); Writing‐review & editing (supporting). **Thierry Tran:** Conceptualization (supporting); Writing‐review & editing (lead). **John Belalcazar:** Writing‐review & editing (lead). **Lucienne Desfontaines:** Writing‐review & editing (equal). **Fabrice Davrieux:** Conceptualization (lead); Writing‐original draft (lead); Writing‐review & editing (supporting).

## Conflict of interest

The authors declare no conflict of interest in this work.

## Ethical guidelines

Ethics approval was not required for this research.

### Peer review

The peer review history for this article is available at https://publons.com/publon/10.1111/ijfs.14773.

## Data Availability

Data sharing not applicable to this article as no data sets were generated or analysed during the current study.
